# Semi-Supervised Deep Kernel Active Learning for Material Removal Rate Prediction in Chemical Mechanical Planarization

**DOI:** 10.3390/s23094392

**Published:** 2023-04-29

**Authors:** Chunpu Lv, Jingwei Huang, Ming Zhang, Huangang Wang, Tao Zhang

**Affiliations:** 1 Department of Automation, Tsinghua University, Beijing 100084, China; lvcp16@mails.tsinghua.edu.cn (C.L.); jwei_huang@foxmail.com (J.H.); hgwang@tsinghua.edu.cn (H.W.); 2 College of Engineering and Physical Sciences, Aston University, Birmingham B4 7ET, UK; m.zhang21@aston.ac.uk

**Keywords:** semi-supervised regression, active learning, deep kernel learning, virtual metrology, phase partition, phase match

## Abstract

The material removal rate (MRR) is an important variable but difficult to measure in the chemical–mechanical planarization (CMP) process. Most data-based virtual metrology (VM) methods ignore the large number of unlabeled samples, resulting in a waste of information. In this paper, the semi-supervised deep kernel active learning (SSDKAL) model is proposed. Clustering-based phase partition and phase-matching algorithms are used for the initial feature extraction, and a deep network is used to replace the kernel of Gaussian process regression so as to extract hidden deep features. Semi-supervised regression and active learning sample selection strategies are applied to make full use of information on the unlabeled samples. The experimental results of the CMP process dataset validate the effectiveness of the proposed method. Compared with supervised regression and co-training-based semi-supervised regression algorithms, the proposed model has a lower mean square error with different labeled sample proportions. Compared with other frameworks proposed in the literature, such as physics-based VM models, Gaussian-process-based regression models, and stacking models, the proposed method achieves better prediction results without using all the labeled samples.

## 1. Introduction

Today, semiconductor manufacturing represents the highest level of microfabrication in the world. Wafer production is a key step in semiconductor manufacturing, which includes many complex processes, such as lithography, etching, deposition, chemical mechanical planarization (CMP), oxidation, ion implantation, diffusion, etc. Processes such as etching, deposition, and oxidation result in wafers with uneven surfaces, including higher steps and larger trenches. CMP technology is an important way to build wafer structures.

The material removal rate (MRR) is a critical variable in the CMP process. Each wafer needs to be polished to the target thickness; thus, an accurate MRR estimation is required to accurately set the polishing time. However, the MRR is difficult and costly to measure, and at the same time, it may cause damage to the wafers. In this stage, the MRR relies on engineering experience and actual measurements with a very low frequency (e.g., only one wafer is selected for measurement in one lot, or one wafer is measured at fixed time intervals) as a reference to complete the setup and adjustment.

In industrial production, to accurately estimate an important but difficult-to-measure variable, it is customary to model the relationship between the variable and easily accessible environmental variables, equipment parameters, intermediate variables, etc., and complete the prediction with physical laws or historical data. This process is called virtual metrology (VM). VM consists of two main forms: physics-based models and data-based models. Physics-based models establish mathematical expressions by analyzing the physical and chemical reactions of the process. Preston proposed that the MRR is related to pressure and the relative velocities of wafers and polishing pads and established the Preston equation [1]. Teh and Chen et al. investigated the dressing process of polishing pads by designing pad simulation modules to investigate the effect of key geometrics [2]. Shin and Kulkarni et al. tested the effects of different diamond structures on the polishing pad and polishing performance [3]. Liu et al. established the relationship of the MRR of fused silica with the polishing pressure, chemical reagents, and their concentrations, as well as the relative velocity between the wafer and the polishing pad [4].

Some of the existing physics-based models build global-based mathematical expressions by analyzing the physical and chemical reactions of the CMP process. Other models focus on the role of certain variables in the CMP process and the effects on the MRR. However, the CMP process involves so many variables that explicit mathematical expressions and physics-based models can hardly cover all of them. At the same time, the duration of the CMP process is very long. In an operating cycle, the CMP process passes through multiple operating points, dividing the process into multiple phases. The different relationships between variables in different phases also lead to continuous changes in the physical models. The global-based physical models are difficult to describe in detail in terms of process changes. With the continuous development of data science, researchers are increasingly placing emphasis on mining knowledge and information from large amounts of historical data, and therefore, data-based VM models are increasingly favored.

Since individual machine learning models do not suitably fit the CMP process, some studies have used stacking models. Zhao and Huang performed one-hot encoding of the “stage” and “chamber” variables in the feature creation and feature encoding stages to transform the process data into multidimensional information, and the stacking integration model was chosen for the regression [5]. Li and Wu et al. also used a stacking integrated learning regression model with primary learners including random forest (RF), gradient boosting tree (GBT), and extreme random tree (ERT), and meta-learner as extreme learning machine (ELM) and classification and regression tree (CART), and the features in the model included the frequency domain features of the three rotation variables and feature selection using RF [6].

The CMP process runs continuously, and the dynamic prediction of the MRR is necessary. Cai and Feng et al. combined k-nearest neighbors (kNN) and Gaussian process regression (GPR) to dynamically predict the MRR, where kNN searched historical data as reference samples and a multi-task Gaussian process (MTGP) model incorporated the training data and reference sample information [7]. The authors proposed another dynamic prediction model, in which reference samples from historical data were fused with data samples through support vector regression (SVR), and a particle filter (PF) estimated and updated the prediction results and ensured that the model could track changes in the CMP process [8]. Lee and Kim addressed the problem of the degradation of the model’s prediction performance due to process drift and proposed a VM model combining the recurrent neural network (RNN) and the convolutional neural network (CNN), extracting time-dependent and time-independent features with a two-stage training method to alternately update the network weights [9]. The existing stacking models and dynamic prediction models in the literature focus on extracting global features and are insensitive to the phase changes in the CMP process. Therefore, a more adequate feature extraction method is needed.

Several researchers have proposed prediction methods based on a combination of physical models and data models [10]. Yu and Li et al. derived a formula for MRR using a pad and conditioner. This model considers the contact between the polishing pads, abrasives, and wafers, and the polishing pad surface topology term in the formula is estimated using RF [11]. Lim and Dutta proposed that the kinetics and contact between the wafer and polishing equipment are applied based on knowledge obtained a priori and a physical model for wafer classification, and they also proposed classification and regression methods, where the feature extraction is obtained by downsampling and expanding the dosage and pressure variables [12]. These methods add physical analysis of the CMP process to the data models. However, similar to the physics-based VM model, the physical analysis focuses on some important variables but cannot cover all the variables’ effects on the MRR.

Research on neural networks and deep learning has provided new ideas [13,14]. Lim and Dutta used autoencoder and k-means clustering to determine the feature space of the training samples and combined the reconstruction loss of the encoder–decoder and the clustering error to form a loss function [15]. Xia and Zheng et al. applied hypergraph convolutional networks to MRR prediction [16]. In the data preprocessing stage, the piecewise aggregate approximation (PAA) method was used for time series alignment and dimensionality reduction. In the prediction stage, a hypergraph model was used to represent the complex relationships between the devices and variables. Maggipinto et al. used deep learning methods to model images of optical emission spectra (OES) with spatial and temporal evolution to build VM models of visual 2D data [17]. Wu et al. designed a VM model combining CNN and Gaussian process regression (GPR) to address the mismatch between VM models and features, using CNN for feature extraction and GPR for the prediction of quality variables [18]. The deep learning methods can fully explore the process features and the relationships between variables but require a large amount of data; otherwise, there is a risk of overfitting. However, due to the difficulty of measuring the MRR, there are less data for the CMP process.

There are some points for improvement regarding the established MRR prediction methods. First, the CMP process is a batch process, and its process data have three dimensions: the batch, variable, and time. Meanwhile, the CMP process involves several phases, and the work points change over time. The different batches and phases are not equal in duration. Most of the existing prediction methods extract the overall features of the process and ignore the phase features. Second, feature extraction with machine learning only involves statistical features, as well as other features such as the duration and nearest neighbors, and the mining of deep features and deep information in the CMP process data is not sufficient. Third, it is worth noting that a large number of wafers lack an MRR and become unlabeled samples because the MRR is not easy to measure. If labeled samples alone are used to build VM models, this will cause information wastage.

In this paper, a semi-supervised deep kernel active learning (SSDKAL) method is proposed for MRR prediction, with the following main contributions:

First, the semi-supervised active learning framework can fully exploit and utilize information regarding unlabeled samples. In this framework, semi-supervised regression (SSR) incorporates the uncertainty of the unlabeled samples into the loss function, while active learning (AL) can compensate for the weakness of the insufficient representativeness and diversity of samples in SSR. Second, clustering-based phase partition and phase-matching algorithms are used to extract phase features. Third, neural networks provide more in-depth identification and fitting of complex multi-phase processes through complex network connections. Our experiments based on CMP process data show that the proposed method achieves good results in terms of the prediction accuracy for the MRR. In actual production, many batch processes have the characteristics of multiple phases and unequal lengths, and there are a large number of unlabeled samples. Therefore, the proposed method can be extended to these processes. In the feature extraction stage, feature data are extracted using phase partition and phase matching, and in the prediction stage, a combination of DKL, SSR, and AL are used for prediction.

The rest of this paper is organized as follows. Chapter 2 presents the related works, including deep kernel learning and active learning regression. Chapter 3 describes the procedure of the SSDKAL method in detail. Chapter 4 presents the experimental results and the discussion. Chapter 5 concludes the paper.

## 2. Related Works

### 2.1. Deep Kernel Learning

Gaussian process regression (GPR) was first proposed by Rasmussen [19]. As an efficient statistical learning method, GPR computes optimal hyperparameters by learning prior functions of historical data to obtain predictive models. With its good interpretability, GPR is widely used in system modeling for industrial processes, especially complex systems with high dimensionality, small samples, and nonlinear characteristics [20]. GPR can provide rich statistical representation, accurate prediction, and new insights into modeling using large datasets.

A Gaussian process (GP) is a stochastic process in which observations appear in a continuous domain (e.g., time or space). In a Gaussian process, each point in the continuous input space is associated with a Gaussian-distributed random variable. Any finite number of random variables obey the joint Gaussian distribution, denoted as Equation (1):

(1) $$f\left(X\right)∼N(\mu \left(X\right),\Sigma_{X,X})$$

where μ(X) denotes the mean function and $\Sigma_{X,X}=\left[\sigma_{ij}\right]=\left[\sigma (x_{i},x_{j})\right]$ denotes the covariance function.

GPR is a typical nonparametric model based on a Bayesian framework. Suppose that we obtain a set of *n* independently and identically distributed observation samples that constitute the input matrix $X={\left(x_{1},x_{2},\cdots ,x_{n}\right)}^{T},x_{i}\in R^{d}$, where *d* denotes the dimensionality of the input samples. The output values corresponding to the samples constitute the vector $y={\left(y_{1},y_{2},\cdots ,y_{n}\right)}^{T},y_{i}\in R$. Let the matrix of the test samples to be $X^{*}={\left(x_{1}^{*},x_{2}^{*},\cdots ,x_{m}^{*}\right)}^{T}$. From the definition and properties of GPR, it is known that the training samples and the test samples obey the joint Gaussian distribution, and y and the model prediction of the test samples $f(X^{*})$ obey the joint Gaussian distribution, as shown in Equation (2):

(2) $$\left[\begin{array}{c} y\\ f(X^{*}) \end{array}\right]∼N\left(\left[\begin{array}{c} \mu (X)\\ \mu (X^{*}) \end{array}\right],\left[\begin{array}{cc} \Sigma_{X,X}+\sigma^{2}I & \Sigma_{X,X^{*}}\\ \Sigma_{X^{*},X} & \Sigma_{X^{*},X^{*}} \end{array}\right]\right)$$

where μ(X) and $\mu (X^{*})$ are the mean vectors of the training and test samples, respectively, $\Sigma_{X,X}$ and $\Sigma_{X^{*},X^{*}}$ are the self-covariance matrices of the training and test samples, respectively, and $\Sigma_{X,X^{*}}=\Sigma_{X^{*},X}^{T}$ is the covariance matrix between the training and test samples. This can be deduced as shown in Equations (3)–(5):

(3) $$f\left(X^{*}\right)|y,X,X^{*}∼N\left(\mu_{f},\Sigma_{f}\right)$$

(4) $$\mu_{f}=\mu \left(X^{*}\right)+\Sigma_{X^{*},X}{\left(\Sigma_{X,X}+\sigma^{2}I\right)}^{-1}\left(y-\mu \left(X\right)\right)$$

(5) $$\Sigma_{f}=\Sigma_{X^{*},X^{*}}-\Sigma_{X^{*},X}{\left(\Sigma_{X^{*},X^{*}}+\sigma^{2}I\right)}^{-1}\Sigma_{X,X^{*}}$$

where $\mu_{f}$ is the predicted mean vector, i.e., the predicted value of the GPR model, while $\Sigma_{f}$ denotes the error region of the predicted output.

Assuming that the parameters of $\Sigma_{X,X}$ are θ, and $\Sigma_{X,X}$ is replaced by $\Sigma_{\theta }$, while the input data are considered to have completed normalization with a mean of 0, according to Equations (3)–(5), y conforms to a Gaussian distribution with a mean of 0 and a variance of $\Sigma_{\theta }+\sigma^{2}I$. The probability distribution function of y is shown in Equation (6):

(6) $$p\left(y|\theta ,X\right)=\frac{1}{{\left(2\pi \right)}^{\frac{n}{2}}\left|\Sigma_{\theta }+\sigma^{2}I\right|}exp\left(-\frac{1}{2}y^{T}{\left(\Sigma_{\theta }+\sigma^{2}I\right)}^{-1}y\right)$$

After taking the logarithm, we can obtain Equation (7):

(7) $$\log p\left(y|\theta ,X\right)\propto -\left[y^{T}{\left(\Sigma_{\theta }+\sigma^{2}I\right)}^{-1}y+\log\left|\Sigma_{\theta }+\sigma^{2}I\right|\right]$$

Wilson and Hu et al. introduced deep networks into the Gaussian process by replacing the kernel and proposed deep kernel learning (DKL) method, which combines deep learning with existing kernel functions using techniques such as deep feedforward, convolutional architectures, and structure utilization algebra to transform deep architectures into inputs for spectral hybrid-based kernels [21]. The deep kernel is as shown in Equation (8):

(8) $$\sigma \left(x_{i},x_{j}|\theta \right)\to \sigma \left(g\left(x_{i},w\right),g\left(x_{j},w\right)|\theta ,w\right)$$

where g(x,w) is a nonlinear mapping of the deep network, and *w* is a network parameter; therefore, the parameters of DKL become η=(θ,w). Similarly, the deep kernel model learns the parameters by maximizing the log marginal likelihood function *L* of the Gaussian process, in which the chain rule is used to calculate the derivatives of the log marginal likelihood function with respect to the parameters and update the parameters through backpropagation. The derivatives are calculated as shown in Equations (9) and (10):

(9) $$\frac{\partial L}{\partial \theta }=\frac{\partial L}{\partial \Sigma_{\eta }}\frac{\partial \Sigma_{\eta }}{\partial \theta }$$

(10) $$\frac{\partial L}{\partial w}=\frac{\partial L}{\partial \Sigma_{\eta }}\frac{\partial \Sigma_{\eta }}{\partial g(x,w)}\frac{\partial g(x,w)}{\partial w}$$

where $\Sigma_{\eta }$ is the depth kernel covariance matrix, and the KISS-GP matrix can be used instead in the calculation.

### 2.2. Active Learning Regression

The purpose of active learning (AL) is to manually label fewer samples from unlabeled samples to achieve the target accuracy when both labeled and unlabeled samples are available. AL is an iterative framework that actively selects the most useful samples in the unlabeled sample set during each iteration and passes them on to experts for labeling, after which they are added to the training set and the current model is updated. AL greatly reduces the amount of data needed to train the model by prioritizing the expert labeling, thus reducing the cost while improving accuracy [22].

In AL, the strategy of selecting valuable unlabeled samples is the core of the algorithm. For active learning classification (ALC), there are numerous strategies, while there are fewer strategies for active learning regression (ALR) [23]. Wu proposed three criteria for pool-based ALR strategies, namely, informativeness, representativeness, and diversity. Informativeness indicates that the queried samples need to contain rich information, which, after labeling, can improve the prediction performance of the model. Representativeness can be measured based on the number of nearest neighbors of the sample, and if the number is high, the sample is more representative. Diversity indicates that the samples need to be dispersed throughout the input space rather than concentrated in a category or a region [23].

Seung and Opper, et al. proposed the query by committee (QBC) method for ALC, in which a set of classifiers are trained on labeled data and then predict unlabeled data, from among which the samples with the most divergent committee members are selected for labeling [24]. Freund et al. demonstrated that the method is applicable when the samples are not labeled as discrete [25]. Krogh and Vedelsby defined the ambiguity of a sample in terms of prediction variance in a committee composed of neural networks and queried the sample label with the largest variance [26]. RayChaudhuri and Hamey used a similar approach but employed bagging in the design of the models [27].

Cai et al. proposed an AL framework for expected model change maximization (EMCM), which aims to select samples from an unlabeled sample set that maximizes the change in the model before and after labeling [28]. The model change is quantified according to the difference in the model parameters before and after the addition of the given sample.

Yu and Kim proposed a passive sampling technique for ALR to identify informative samples based on their geometric features in the feature space [29]. They argued that the samples selected for queries based on loss functions have relatively large errors and are likely to be noisy. They proposed four passive sampling methods, including the Grid approach, k-center algorithms, greedy sampling (GS) algorithm, and incremental k-medoids algorithm.

Wu and Lin et al. argued that the above GS algorithm takes into account the diversity of samples in the input space and proposed two other GS methods. The first method, GSy, takes into account the diversity of samples in the output space, and the second method, iGS, takes into account the diversity of samples in both the input and output spaces [30].

Wu proposed a new ALR method using passive sampling that incorporates the idea of clustering in the initialization and iteration while considering informativeness, representativeness, and diversity. The method can complete the process of ALR without a single labeled sample. This method can also be combined with QBC, EMCM, and greedy sampling [23].

## 3. Methods

### 3.1. Feature Extraction

The data for the CMP process have three dimensions: the batch, variable, and time. Usually, the data of a batch represent the production process of a wafer, i.e., the data matrix of variable×time, corresponding to an MRR value. The main processing method for 3D data is to expand the data in the variable direction or batch direction and recover them as 2D data. Multiple principal component analysis (MPCA) and multiple partial least squares (MPLS) are then used for early batch process statistical monitoring and regression analysis, respectively [31,32].

Due to variations in the equipment and environment, the duration of each wafer is different. The above-mentioned MPCA, MPLS, and most other methods are not feasible for a CMP process that is unequal in length. At the same time, the CMP process is divided into several phases, and the variable relationships are different in different phases, with different operating points of the devices. The extraction of global features will only lead to the loss of information. In this paper, we adopt a feature extraction method based on phase partition and phase matching.

In phase partition, a combination of wrapped k-means (WKM) [33] and the phase partition combination index (PPCI) are used. The dimensionality of each wafer data matrix is different because the wafers have different operation times. The purpose of phase partition is to obtain more accurate phase features; thus, phase partition is performed separately for each wafer. The data of the *I*-th batch are $X={\left(x_{1},x_{2},\cdots ,x_{K_{I}}\right)}^{T}$, where $K_{I}$ denotes the sampling length, i.e., the number of sample points contained in the batch, and $x_{k}\in R^{J},k=1,2,\cdots ,K_{I}$, where *J* is the number of variables contained in each sample point. The basic idea of phase partition is that the sample vectors in the batch are clustered, and different categories correspond to different phases. However, it is worth noting that the process data need to satisfy the temporal order constraint. Given the number of clusters, i.e., the number of phases *C*, the batch data are initially divided according to the cumulative error, and then the sampling points are moved. Here, the sum of squared error (SSE) is used to calculate the clustering error and acts as the basis for the sampling point movement. The SSE is calculated as shown in Equation (11):

(11) $$SSE=\sum_{c=1}^{C}\sum_{x_{k}\in Phase\;c}{\left(x_{k}-s_{c}\right)}^{T}\left(x_{k}-s_{c}\right)$$

where $s_{c}$ is the center point of the *c*-th phase. The following rule is used for sample point movement, when the movement condition is met, the first half of the sample points of each phase can only move to the previous phase, and the second half can only move to the latter phase. If a sample point does not meet the move condition, the remaining sample points in that half will no longer move. After a sample point $x_{k}$ moves from phase *c* to phase *b*, the centroids will change, as shown in Equations (12) and (13):

(12) $${\hat{s}}_{c}=s_{c}-\frac{x_{k}-s_{c}}{l_{c}-1}$$

(13) $${\hat{s}}_{b}=s_{b}+\frac{x_{k}-s_{b}}{l_{b}+1}$$

where $l_{c}$ and $l_{b}$ are the numbers of sampling points in phase *c* and phase *b* before the move, respectively. After that, the SSE is recalculated, and if the SSE decreases, $x_{k}$ will be moved to phase *b*. Otherwise, the move will be rejected.

The CMP process contains stable phases with small changes in variable relationships and transition phases with fast changes in variable relationships. In the WKM algorithm, given the number of clusters *C*, a wafer can be divided into *C* phases, but the effectiveness of the partition is related to the size of *C*. When *C* is larger, the partition is detailed, and more transition phases are divided. When *C* is small, more transition phases are merged into the stable phases. We weigh the phase number *C* and the clustering error SSE and use PPCI for the metric. With the increase in *C*, PPCI shows a trend of decreasing and then increasing. The phase number corresponding to the smallest PPCI is chosen as the phase partition number of the wafer.

Due to the presence of PPCI, the optimal phase number differs from wafer to wafer. In the CMP process, many wafers have more transition phases with drastic changes, and the PPCI still decreases when the cluster number is large. The ultimate goal of phase partition is to extract the features of key phases; therefore, the phases of different wafers need to be matched. First, we must eliminate the effect of transition phases. Given a minimum length $L_{S}$, the phase with a number of sampling points no lower than $L_{S}$ is considered as a stable phase; otherwise, it is a transition phase. Next, the standard stable phase number (SSN) and the standard stable phase center (SSPC) are determined. For all wafers, the plural of the stable phase number is selected as SSN. All wafers with stable phase numbers equal to SSN are selected, the mean value of the centroid of each stable phase is calculated, the wafers far from the mean value are discarded, and the process is iterated to finally obtain the SSPC.

After that, all wafers that have completed phase partition are matched with the SSPC. The basic method of phase matching is to calculate each phase center and identify the SSPC with the closest distance to it. However, at the same time, the time series constraint needs to be maintained. Two phases with backward and forward orders of time cannot be matched with SSPCs with opposite time orders. The optimization problem of phase matching is as shown in Equation (14):

(14) $$\begin{array}{cc} min & \sum_{h=1}^{SSN}\left[dist\left(s_{h}^{*},{\tilde{s}}_{h}^{i}\right)-\rho {\tilde{L}}_{h}\right]\\ s.t. & {\tilde{L}}_{h}=end_{h}^{i}-start_{h}^{i}\\ \; & {\tilde{s}}_{h}^{i}=\frac{1}{{\tilde{L}}_{h}}\sum_{l=start_{h}^{i}}^{end_{h}^{i}}x_{l}\\ \; & start_{h+1}^{i}\geq end_{h}^{i},h=1,2,\cdots ,SSN-1\\ \; & dist\left(s_{h}^{*},s_{t}^{i}\right)\leq \theta ,t=J_{h},J_{h}+1,\cdots ,J_{h}+T_{h}-1 \end{array}$$

where *i* denotes the matching process of the *i*-th wafer, $s_{h}^{*}$ denotes the *h*-th SSPC, and ${\tilde{s}}_{h}^{i}$ denotes the *h*-th phase center of the *i*-th wafer after phase matching is completed. $start_{h}^{i}$, $end_{h}^{i}$, and ${\tilde{L}}_{h}$ denote the start point, end point, and the length of the *h*-phase after the matching, respectively. θ is the distance threshold of the two centers, ρ denotes the balance parameter of the distance and phase length, and $J_{h}$ and $T_{h}$ denote the starting and ending phases of the original wafer that match the *h*-th standard phase. We use a greedy algorithm to continuously compare the distance between the center of the phases and the SSPC to complete the phase matching.

### 3.2. Semi-Supervised Deep Kernel Active Learning

It is worth noting that, due to the sampling detection of the MRR, a large number of samples are missing the MRR. Attention should be paid to semi-supervised regression (SSR) methods using both labeled and unlabeled samples. Kostopoulos et al. divided SSR into four categories: semi-supervised kernel regression, multi-view regression, graph regularization regression, and semi-supervised GPR [34]. Among them, the semi-supervised GPR model consists of two components in the log-likelihood function: the joint distribution of independent and dependent variables with labeled samples based on the requested parameters, and the joint distribution likelihood function with unlabeled samples based on the requested parameters. The parameter value that maximizes the log-likelihood function is obtained via derivation [35,36,37].

The semi-supervised deep kernel learning (SSDKL) method combining deep learning and the Gaussian process uses the deep neural network (DNN) as a kernel function, and the optimization objective function includes both the log-likelihood function and the prediction variance of the unlabeled samples, the latter being used as a regularization term to suppress the overfitting of the model [38]. The loss function is adjusted to Equation (15):

(15) $$L_{SS}\left(\eta \right)=\frac{1}{L}L_{likelihood}\left(\eta \right)+\frac{\alpha }{U}L_{uncertainty}\left(\eta \right)$$

where α is the equilibrium parameter controlling the two types of losses.

In SSR, information on unlabeled samples is captured by adding the prediction variance as the regularization term of the loss function. In contrast toSSR based on co-training [39], the unlabeled samples and pseudo-labels in SSDKL are not added to the labeled sample set, but by changing the loss function, they have an impact on the parameters of the model. The unlabeled samples provide an informative basis for model improvement but do not take into account the representativeness and diversity. At the same time, the small number of labeled samples in SSR are likely to be unevenly distributed and cannot fully cover the overall distribution of the samples. Active learning (AL) can compensate for this weakness. AL allows the model to select the important samples itself, thus obtaining a higher performance with less training samples.

Considering the three sample selection criteria, this paper uses a sample query strategy based on a combination of query by committee (QBC) and greedy sampling (GS).

Suppose that there are *N* samples in the training set, constituting $X={\left\{x_{i}\right\}}_{i=1}^{N}$, where the set of labeled samples $X_{L}={\left\{x_{i}\right\}}_{i=1}^{L}$ contains *L* labeled samples, and the set of unlabeled samples $X_{U}={\left\{x_{i}\right\}}_{i=L+1}^{L+U}$ contains *U* unlabeled samples, L+U=N. According to QBC, *M* regressors are trained according to $X_{L}$, and the prediction variance of the unlabeled samples is as shown in Equation (16):

(16) $$s_{i}^{QBC}=\frac{1}{M}\sum_{j=1}^{M}{\left(y_{i}^{j}-{\bar{y}}_{i}\right)}^{2}$$

where ${\bar{y}}_{i}$ is the average of the predicted values of the *M* regressors for the *i*-th unlabeled sample. QBC selects the sample with the largest prediction variance for the query. Here, the prediction variances of all unlabeled samples are sorted from largest to smallest, and the set of unlabeled samples with the largest variance are selected and denoted as $X_{QBC}$.

Next, from the set $X_{QBC}$, GS selects the sample farthest from the existing labeled sample for the query. The distance is calculated as shown in Equation (17), and the unlabeled sample corresponding to the largest $s_{i}^{GS}$ will become the queried sample.

(17) $$s_{i}^{GS}=\underset{x_{l}\in X_{L}}{min}∥x_{i}-x_{l}∥$$

The semi-supervised deep kernel active learning (SSDKAL) method is proposed. In each round of iterations, a combination of QBC and the GS sample selection strategy are used. QBC focuses on the performance improvement of the model with respect to the query sample, i.e., informativeness, while GS takes into account the representativeness and diversity. The training process starts by training a set of SSDKL regressors using a small number of labeled samples. All regressors make predictions based on the unlabeled samples, select a set of samples with the largest variance, and choose a subset of unlabeled samples from this set, which are farthest from the existing labeled samples. The sample labels in this subset are queried and added to the labeled sample set. The learning, prediction, and sample selection process are repeated until the accuracy constraint or the upper limit of the query capability is reached.

The algorithm of SSDKAL is shown in Algorithm 1. Without loss of generality, we assume that there are only two regressors in the AL committee, $f_{A}\left(x|\eta_{A}\right)$ and $f_{B}\left(x|\eta_{B}\right)$.
**Algorithm 1** SSDKAL**Input:** After phase partition and phase matching, the input data *X*, which have completed phase feature extraction, feature selection, and normalization, include the labeled dataset $X_{L}=\left\{\left(x_{1},y_{1}\right),\cdots ,\left(x_{L},y_{L}\right))\right\}$, unlabeled dataset $X_{U}=\left\{\left(x_{L+1}\right),\cdots ,\left(x_{L+U}\right))\right\}$, learning rate $l_{r}$, unlabeled sample loss term weight parameter α, maximum number of iterations $Max_{ep}$, maximum AL query sample number $M_{AL}$, and error threshold ϵ.**Output:** Final model parameters $\eta_{A}^{*}=\left\{\theta_{A}^{*},w_{A}^{*}\right\}$, $\eta_{B}^{*}=\left\{\theta_{B}^{*},w_{B}^{*}\right\}$.  1:Initializing model parameters $\eta_{A}^{0}=\left\{\theta_{A}^{0},w_{A}^{0}\right\}$, $\eta_{B}^{0}=\left\{\theta_{B}^{0},w_{B}^{0}\right\}$.  2:The query number of AL qey=0.  3:**while** $qey\leq M_{AL}$ **do**  4: Construct semi-supervised deep kernel regression models $f_{A}\left(x|\eta_{A}^{0}\right)$, $f_{B}\left(x|\eta_{B}^{0}\right)$ using $X_{L}$.  5: Iteration number i=0.  6: **while** $i\leq Max_{ep}$ **do**  7:   Calculate the loss function of the models based on the labeled and unlabeled datasets.  8:   $L_{labeled}^{i}=\frac{1}{L}\sum_{x\in X_{L}}L_{likelihoood}\left(\eta_{A/B}^{i}\right)$  9:   $L_{unlabeled}^{i}=\frac{1}{U}\sum_{x\in X_{U}}L_{uncertainty}\left(\eta_{A/B}^{i}\right)$10:   $L_{SS}^{i}=L_{labeled}^{i}+L_{unlabeled}^{i}$11:   Compute the derivative of the loss function $L_{SS}^{i}$ with respect to the parameter $\eta_{A/B}^{i}=\left\{(\theta_{A/B}^{i},w_{A/B}^{i})\right\}$ and update the parameter using stochastic gradient descent (SGD).12:   $\theta_{A/B}^{i+1}\leftarrow SGD\left(L_{SS}^{i},l_{r},\theta_{A/B}^{i}\right)$13:   $w_{A/B}^{i+1}\leftarrow SGD\left(L_{SS}^{i},l_{r},w_{A/B}^{i}\right)$14:   **if** $abs(L_{SS}^{i},L_{SS}^{i-1})\leq \epsilon$ **then**15:    End the training.16:   **end if**17:   i=i+118: **end while**19: Calculate the outputs of the models for unlabeled data, $f_{A}\left(x|x\in X_{U}\right)$, $f_{B}\left(x|x\in X_{U}\right)$.20: Sort ${\left(f_{A}\left(x\right)-f_{B}\left(x\right)\right)}^{2}$ and take the first *K* maxima to form the unlabeled data subset.21: Calculate the sample farthest from $X_{L}$ in the subset, query its label, add it to the labeled dataset $X_{L}$, and remove it from $X_{U}$.22: qey=qey+123:**end while**24:**return**: Model parameters $\eta_{A}^{*}=\left\{\theta_{A}^{*},w_{A}^{*}\right\}$, $\eta_{B}^{*}=\left\{\theta_{B}^{*},w_{B}^{*}\right\}$

**Figure 1:**
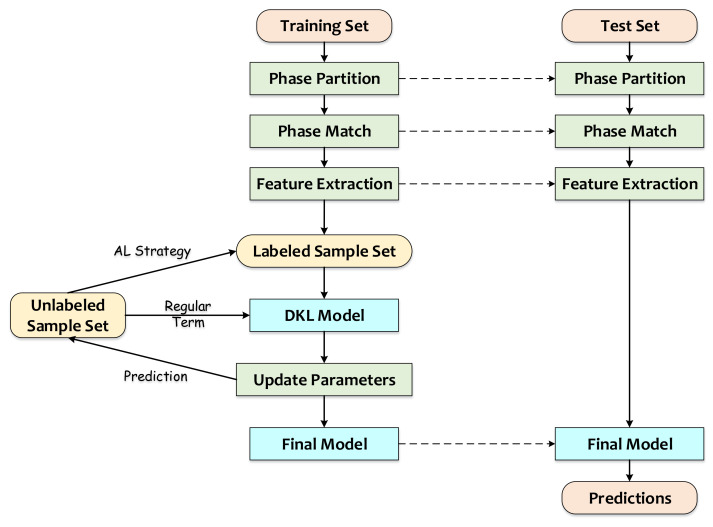
Algorithm flow of SSDKAL.

Figure 1 shows the algorithm flow of SSDKAL. First, the training samples pass through the steps of phase partition, phase matching, and feature extraction to obtain the initial dataset, which includes the labeled sample set and the unlabeled sample set. According to the SSDKAL algorithm, the DKL model is built using the labeled sample set, and the model parameters are updated based on the log marginal likelihood function of the labeled samples and the uncertainty of the unlabeled samples. The model predicts the outputs of the unlabeled samples and selects the samples that satisfy the informativeness, representativeness, and diversity according to the AL strategy of combining QBC and GS, querying the labels, and adding them to the labeled sample set. For the test set, the phase partition, phase matching, and feature extraction are similar to the training set. After the phase partition for each wafer, the SSN and SSPC obtained from the training set are applied to complete the phase matching, and the final model is used for the prediction.

## 4. Experiments and Discussion

### 4.1. Datasets

The dataset is from the 2016 Prognostics and Health Management (PHM16) Data Challenge and contains a set of CMP process data. The dataset is divided into a training set, a validation set, and a test set, each of which contains process data from several wafers. The number of sampling points for each wafer is between roughly 200 and 400, and each sampling point contains 25 variables, as shown in Table 1. Among them, 6 variables are device and wafer information, and the remaining 19 variables are process variables, which are divided into 5 groups, including usage, pressure, flow, rotation, and status. Each wafer is identified based on two variables, “Wafer_ID” and “Stage”, and corresponds to an MRR value.

**Figure 2:**
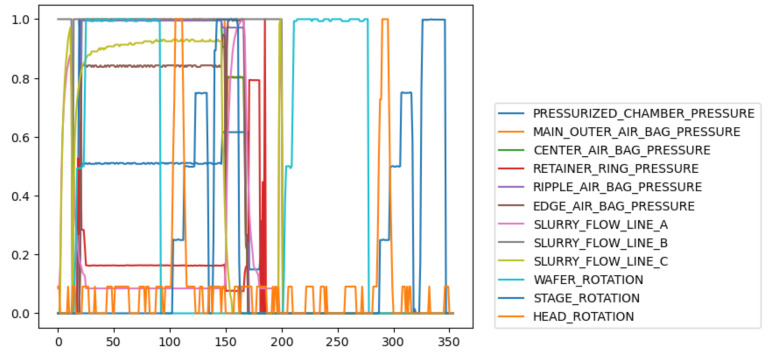
Different variables of a wafer.

Figure 2 shows the trajectories of some variables of a wafer, including six pressure variables, three flow variables, and three rotation variables. It can be seen these variables have different relationships in different phases, which also indicates that the CMP process is a multi-phase process.

All wafers can be divided into three modes based on the “Chamber” and “Stage”. Table 2 shows the distribution of three modes, including the chamber, stage, and the range of the MRR, as well as the numbers of samples in the training set, validation set, and test set.

### 4.2. Feature Extraction

For each wafer, we perform phase partition using the WKM-PPCI algorithm. During the CMP process, different variables have different trends. Among them, the usage variables have an increasing trend and do not conform to the phase change characteristics. The pressure, flow, and rotation variables have phase change characteristics, and the pressure variables have more obvious changes and coincide with the switching of chambers at certain change time points. Therefore, six pressure variables are used for clustering and phase partition.

Given the phase number *C*, integers of $\left[4,15\right]$ are taken, and the number corresponding to the smallest PPCI is chosen as the phase number of this wafer. After the phase partition, the minimum number of sampling points $L_{S}=5$ contained in the stable phase is selected according to the phase alignment algorithm to distinguish the stable phases and the transition phases for each wafer. The plural of all the wafers’ stable phases is chosen as the SSN, and in this experiment, the SSN = 5. The SSPC is calculated iteratively, and the phases of all the wafers are matched with the SSPC using a greedy algorithm. Finally, all the wafers are divided into five phases.

**Figure 3:**
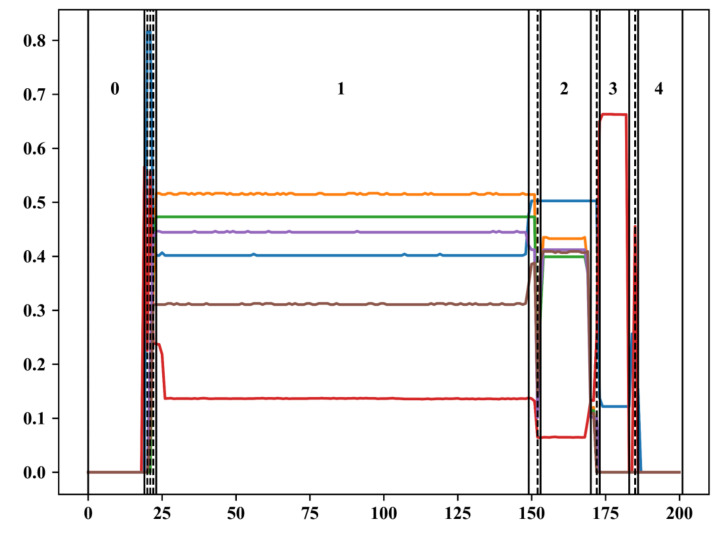
Phase partition and phase match of a wafer.

Figure 3 shows the phase partition and phase match of a wafer. In Figure 3, the horizontal axis indicates the time order of the sampling points, and the vertical axis indicates the normalized variable values. The colored solid lines indicate the variation of the 6 pressure variables along time. The vertical dashed line indicates the optimal phase partition results after using the phase partition algorithm with the combination of WKM and PPCI. It can be seen that due to the presence of the transition phases, the phase partition is very detailed in the transition phases, and the optimal number of phases is large, which is 15 in the case of this wafer. The solid line on the vertical axis and the number between the solid lines indicate the results of the phase match. After the phase match, 15 phases are matched with 5 standard stable phase centers.

As shown in Table 3, for each phase, the pressure, flow, and rotation variables coincide with the change in the phases, and five statistical features are extracted, including the mean, standard deviation (std), median, peak-to-peak (PtP), and area under the curve (AUC). The status variable has only two states of 0 and 1, the median and PtP are meaningless, and the mean and AUC are redundant variables; only the mean and std features are retained. The usage variable is an incremental variable independent of the phase change, and only the initial value feature is extracted. In addition, the start time and duration are extracted from the timestamp variable. Seventy variables are extracted for each phase, forming a large feature set. The filtered feature selection method is used, which can filter out single numerical features, low-variance features, high-linear-correlation features, etc.

### 4.3. Prediction Results of Different Regression Models

After obtaining the phase features, we apply different models to the PHM16 Dataset. Table 4 and Table 5 show the prediction results of different types of regression models for Mode I and Mode II. The prediction results of each regression model are the average predicted values obtained after running several experiments. In this paper, the criterion for comparing the prediction results is the mean square error (MSE), as shown in Equation (18):

(18) $$MSE=\frac{1}{N_{test}}\sum_{i=1}^{N_{test}}{\left({\hat{y}}_{i}-y_{i}\right)}^{2}$$

where $N_{test}$ is the number of samples in the test set, and ${\hat{y}}_{i}$ and $y_{i}$ are the prediction and true values of the *i*-th test sample, respectively. The bold numbers in Table 4 and Table 5 indicate the prediction error values with the best prediction performance. In our experiments, we chose four supervised regression models, including k-nearest neighbor (kNN) regression [40], support vector regression (SVR) [41], ExtraTree (ET) [42], and Gaussian process regression (GPR), and three SSR models, including Coreg-kNN, Coreg-SVR, and SSDKL, in addition to the proposed method, SSDKAL. “Global Features” indicate that the models do not use phase partition or phase match methods, and only global features are extracted from the process data. The regression models used for the “Global Features” are four supervised regression models, including kNN, SVR, ET, and GPR.

The models use multiple experiments to obtain the mean value as the prediction results. Among them, Coreg is a semi-supervised method based on co-training and involves multiple regression models [43]. Coreg-kNN and Coreg-SVR denote the regression models of kNN regression and SVR, respectively.

“Label_Ratio” indicates the proportion of labeled samples in the training set, and the formula is shown in Equation (19):

(19) $$Label\_Ratio=\frac{L}{L+U}\times 100\%$$

where *L* and *U* denote the numbers of labeled samples and unlabeled samples in the training set, respectively.

In the supervised regression models, the number of nearest neighbors of kNN is 15, the penalty parameter of SVR is 1.0, the minimum number of samples of split points in ET is 2, and the minimum number of samples of leaf nodes is 1. In SSR, the numbers of nearest neighbors of the two kNN models chosen by Coreg-kNN are 10 and 15, and the penalty parameters of the two SVR models chosen by Coreg-SVR are 0.1 and 1.0, respectively. The front-end network of SSDKL uses multiple fully connected layers, the parameter of the activation function LeakyReLU is 0.2, and the optimizer chooses stochastic gradient descent (SGD) with a learning rate of 0.01. The main parameters of SSDKAL are the same as those of SSDKL, but multiple models need to be included as committees of AL, and the main difference between the models is the number of layers and nodes of the fully connected layers.

The results in Table 4 and Table 5 show that the prediction errors of the different models basically show a decreasing trend as the proportion of labeled samples increases. This is an expected result, because the increase in the amount of labeled data causes the models to obtain more accurate information, and the models can fit the real variable relationships more accurately. However, there are also some models for which the prediction results fluctuate as the proportion of labeled samples increases. On the one hand, supervised regression is more prone to fluctuations, because when there is less valid information, the fitting effect of the model is far from the real situation, and an effective prediction model cannot be built. On the other hand, the labeled samples were obtained by random sampling in these experiments; thus, the informativeness and representativeness of the extracted samples vary with different proportions, leading to a situation in which the prediction results fluctuate with the proportion of labeled samples.

In Table 4 and Table 5, the comparisons of the prediction results of the "Global Features" and supervised regression models based on phase features demonstrate the role of the phase features in the proposed framework. For the same regression model and label ratio, the phase features contribute to the accuracy of the prediction results. The proposed phase partition and phase match methods can more fully exploit the trend of the time series data in the CMP process and obtain accurate phase features.

Meanwhile, Table 4 and Table 5 show that the SSDKAL algorithm, which incorporates SSR, GPR, and DKL together with AL, obtains more accurate prediction results than supervised regression and SSR for different labeled sample proportions in both Mode I and Mode II. Compared with the corresponding supervised regression methods (kNN and SVR), the semi-supervised Coreg algorithm, which obtains pseudo-labels of unlabeled data, leads to a substantial improvement in the prediction performance. The addition of DKL leads to a further reduction in the prediction error. The SSDKAL algorithm takes the features initially extracted after phase partition and phase matching and, through information mining via DNN, fitting with a Gaussian process kernel function, and adding the uncertainty information of the unlabeled data, with a very small amount of AL query information, achieves a more accurate MRR prediction.

For comparison, Table 6 shows the prediction results for Mode III. Mode III involves fewer samples, and the multi-phase characteristic is not obvious. For Mode III, we only extracted global features and did not use phase partition or phase matching. The feature number is small. Table 6 shows that the simple model (e.g., kNN) has better prediction results, and the addition of other modules increases the model’s complexity and the risk of overfitting, which prevents the extraction of more feature information. Therefore, the SSDKAL algorithm is more suitable for datasets with a larger data volume and a larger number of features.

Taking Mode I as an example, Table 7 shows the time consumption of different regression models, and the unit of data is seconds. The time for the “Global Features” and supervised regression models is the average of a single run time. The time for the SSR models and SSDKAL is the time for each training round, and the number of training rounds is set to 30. Each round of Coreg-kNN and Coreg-SVR includes the training of multiple models based on labeled data. The models select unlabeled data with high confidence, as well as their pseudo-labels, and add them to the labeled dataset. Each training round of SSDKL consists of training DKL models based on labeled data, predicting unlabeled data, calculating a loss function containing the uncertainty of the unlabeled data, and performing backpropagation. Each round of SSDKAL includes the forward computation and backpropagation of multiple models and query sample selection based on a combination of QBC and GS. Bold numbers indicate the time required by the SSDKAL model. The bold numbers in Table 7 indicate the time consumption of SSDKAL.

The time consumption in Table 7 shows that the simple supervised regression models take very little time but have a lower prediction accuracy. The SSR models and SSDKAL require the prediction, selection, and training of unlabeled samples, and the time required for each training round is long. SSDKAL incorporates deep neural networks for feature extraction and applies the results of multiple regression models to the sample selection strategy of combining QBC and GS, and therefore, the time consumption is higher. However, the SSDKAL method can fully exploit the data features and improve the prediction accuracy by using unlabeled samples.

### 4.4. Ablation Experiments

**Figure 4:**
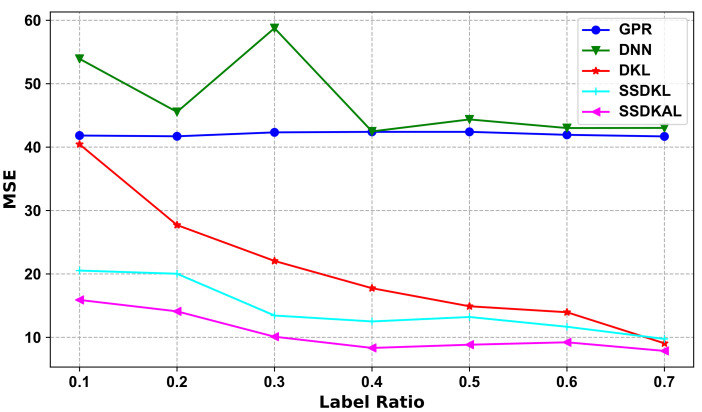
Variations in different models with the label ratio in the ablation experiments based on Mode I.

In the ablation experiments, we split SSDKAL to test the prediction performance of the GPR, DNN, and DKL models separately. DNN uses multiple fully connected layers, with the top-layer features as input to the DKL model. DKL, SSDKL, and SSDKAL optimize all the hyperparameters of the deep kernel and train the network using the log marginal likelihood function to derive the parameters and perform backpropagation. The models are experimentally implemented using GPytorch [44]. The experimental results of the ablation experiments are shown in Table 8 and Figure 4 for Mode I.

As shown in Table 8 and Figure 4, the ablation experiments based on Mode I demonstrate the prediction performance of each model when the proportion of labeled samples is consistent, and the approximate performance ranking is SSDKAL > SSDKL > DKL > GPR > DNN. The GPR model is a typical machine learning model, which requires additional data cleaning and feature engineering. When the modeled data distribution is too complex, the availability of a priori knowledge regarding the data distribution directly determines the accuracy of the data feature engineering, which, in turn, affects the model’s performance. DNN can reduce manual involvement to a greater extent via multi-level feature extraction. DKL gives full play to the advantages of both models by organically combining DNN and GPR and maintains the strong generalization performance with respect to the data, keeping the model performance high while maintaining strong generalization in regard to the data.

The model prediction performance of SSDKAL is best when the proportion of labeled samples is kept the same. In the semi-supervised scenario, pure machine learning has a performance limitation. SSDKAL further enhances the model’s utilization of unlabeled data by incorporating AL methods into the training mechanism of DKL by combining the prediction uncertainty of unlabeled samples with actively learned expert queries and thus improves the comprehensive modeling capacity of the dataset.

### 4.5. Comparisons of Different AL Strategies and Kernel Functions

Table 9 and Table 10 show the prediction performance of the models with different AI query strategies and different kernel functions. The models used in the experiments are all SSDKAL, and the proportion of labeled samples is 0.3.

In Table 9 and Table 10, “QBC + GS” indicates the query strategy using a combination of QBC and GS, i.e., the group of unlabeled samples with the largest prediction variance between different models is selected first; then, that which is farthest from the labeled samples is selected from among them and the label is queried. “QBC”, “GS” indicates that only the QBC or the GS strategy is used, respectively, and only one sample is queried. The “QBC Group” indicates the querying of a group of samples in an epoch using the QBC strategy, in which case the query is faster. The experimental results show that the strategy combining QBC and GS, which considers informativeness, representativeness, and diversity, has the best prediction effect.

RBF, cosine, Matern, and RQ are the four kernel functions in the DKL framework, and the tables show that the cosine kernel has a poor prediction performance. The CMP process is a nonlinear, multi-batch complex production process, and there is no significant correlation between the data features, which is a key factor limiting the performance of the cosine kernel. In comparison, RBF has a better fitting effect. For different application scenarios, the kernel functions and related hyperparameters need to be flexibly selected based on domain knowledge in order to achieve the maximum performance of the model.

### 4.6. Effects of the SSDKAL Method

Table 11 presents the results of existing methods for MRR prediction and compares them with the proposed SSDKAL method. The data shown are the MSE. The ratio of labeled samples for the SSDKAL method is 0.7. SSDKAL achieves good prediction results without using all the labeled samples. In the feature extraction stage, SSDKAL takes into account the unequal length and multi-phase characteristics of the CMP process and extracts detailed process features through phase partition, phase matching, and phase feature extraction. DKL obtains the phase features through deep neural networks and further mines the depth features and the deep associations between variables. SSR adds the uncertainty of unlabeled samples to the loss function. The AL sample selection strategy compensates for the lack of representativeness and diversity of samples in SSR and makes full use of unlabeled sample information.

**Figure 5:**
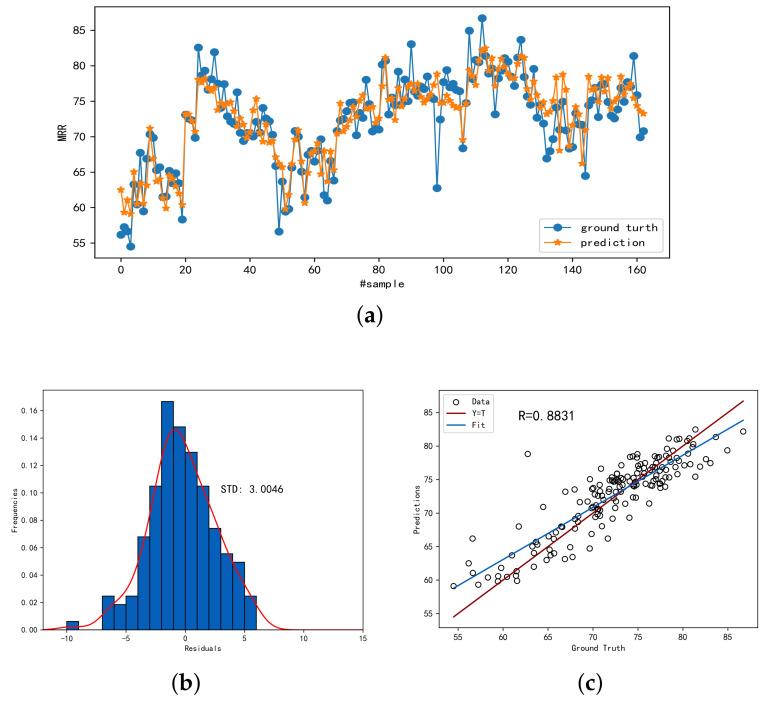
The prediction performance of SSDKAL. (**a**) The predictions and ground truths. (**b**) The histogram distribution of the residuals. (**c**) The linear correlation between the predictions and the ground truths.

Figure 5 demonstrates the prediction accuracy of SSDKAL for a labeled sample ratio of 0.7. Figure 5a shows the predicted values compared with the actual values, Figure 5b shows the distribution of the residuals, and Figure 5c shows the linear analysis of the predicted and actual values. It can be seen that SSDKAL can predict the MRR more accurately.

### 4.7. Limitations

The proposed method, SSDKAL, still has some limitations and drawbacks. Firstly, due to the small number of samples, the query samples for AL are still affected by the problem of insufficient representativeness and diversity. Since the query samples have an incomplete coverage of the overall distribution of the samples, the model’s estimation of the sample distribution is biased, which affects the prediction accuracy. Secondly, in the SSDKAL model, the deep kernel mapping of the Gaussian DKL model uses fully connected layers, which is insufficient for the extraction and mining of the depth features of the sample data. Finally, the AL sample selection strategy is based on the data features of the samples. However, the CMP process is complex, and the utilization of information from the process data is incomplete if knowledge obtained a priori, such as the process mechanism, is completely absent.

## 5. Conclusions

In this paper, we proposed a VM method known as SSDKAL for MRR prediction in the CMP process. In the feature extraction stage, the phase information is fully mined, and the phase features are extracted using phase partition and phase-matching algorithms. In the modeling stage, the combination of deep neural networks and Gaussian process regression enable the deep mining of feature information. Semi-supervised regression and active learning sample query strategies make full use of the information of unlabeled samples. The proposed method was validated based on the CMP process dataset and achieved a better prediction accuracy than supervised regression and semi-supervised regression.

Our future work will focus on applying deep learning methods to data in the CMP process. To date, deep learning has yielded rich research results for both image and time series data, while more accurate and deep feature extraction and processing could be accomplished. The 3D data can be considered as both image and time series data. Therefore, deep learning will play an important role in data research on the CMP process.

## Figures and Tables

**Table 1:** Variables in PHM16 Dataset.

Group	Name	Group	Name
Information	Machine_ID	Pressure	Pressurized_chamber_pressure
	Machine_Data		Main_outer_air_bag_pressure
	Timestamp		Center_air_bag_pressure
	Wafer_ID		Retainer_ring_pressure
	Stage		Ripple_air_bag_pressure
	Chamber		Edge_air_bag_pressure
Usage	Usage_of_backing_film	Flow	Slurry_flow_line_A
	Usage_of_dresser		Slurry_flow_line_B
	Usage_of_polishing_table		Slurry_flow_line_C
	Usage_of_dresser_film	Rotation	Wafer_rotation
	Usage_of_membrane		Stage_rotation
	Usage_of_pressurized_sheet		Head_rotation
Status	Dressing_wafer_status		

**Table 2:** Three modes in PHM16 Dataset.

Mode	Chamber	Stage	MRR	Training	Validation	Test
Mode I	4,5,6	A	[50, 90]	798	185	165
Mode II	4,5,6	B	[50, 110]	815	172	186
Mode III	1,2,3	A	[120, 170]	368	67	72

**Table 3:** Phase features.

Group	Feature	Group	Feature
Timestamp	start_time	Flow	mean
	duration		std
Usage	initial_value		median
Status	mean		peak-to-peak
	std		AUC
Pressure	mean	Rotation	mean
	std		std
	median		median
	PtP		PtP
	AUC		AUC

**Table 4:** Results of different regression models based on Mode I.

Model Class	Models	Label_Ratio						
		0.1	0.2	0.3	0.4	0.5	0.6	0.7
Global features	kNN	46.70	40.94	40.40	39.27	37.16	34.93	33.86
	SVR	49.72	47.87	47.42	46.16	46.45	44.99	44.60
	ET	39.65	38.21	34.79	36.49	26.74	28.51	26.78
	GPR	43.79	42.52	42.75	42.44	42.81	42.19	41.92
Supervied regression	kNN	32.14	25.51	24.89	24.62	23.20	21.54	21.24
	SVR	42.32	38.67	38.11	35.95	35.10	34.16	34.18
	ET	20.96	17.14	20.63	18.69	20.17	13.95	12.40
	GPR	41.83	41.71	42.33	42.41	41.41	41.94	41.69
SSR	Coreg-kNN	20.20	17.34	16.59	16.24	16.44	15.82	16.53
	Coreg-SVR	36.09	29.16	26.99	25.37	24.06	21.96	20.50
	SSDKL	20.53	20.03	13.44	12.51	13.21	11.67	9.75
AL	SSDKAL	**15.90**	**14.10**	**10.10**	**8.33**	**8.84**	**9.22**	**7.86**

**Table 5:** Results of different regression models based on Mode II.

Model Class	Models	Label_Ratio						
		0.1	0.2	0.3	0.4	0.5	0.6	0.7
Global features	kNN	58.45	50.86	45.17	42.44	40.28	38.54	36.77
	SVR	73.70	73.53	73.02	73.03	72.92	72.84	72.75
	ET	40.72	38.95	36.95	28.34	28.14	39.72	28.60
	GPR	78.71	77.30	74.33	73.91	74.03	74.07	74.39
Supervied regression	kNN	51.98	41.93	34.93	32.08	29.54	28.75	27.28
	SVR	71.96	70.30	69.44	67.18	66.21	64.65	64.60
	ET	29.98	21.48	19.46	23.72	17.46	16.98	15.60
	GPR	73.06	73.70	72.73	72.61	72.73	72.92	73.16
SSR	Coreg-kNN	27.81	22.78	19.69	19.77	19.46	19.41	18.46
	Coreg-SVR	55.36	45.41	39.90	36.59	34.53	31.75	29.83
	SSDKL	30.56	20.21	23.25	14.50	10.92	11.78	10.97
AL	SSDKAL	**20.93**	**12.08**	**11.85**	**10.68**	**10.64**	**10.18**	**9.95**

**Table 6:** Results of different regression models based on Mode III.

Model Class	Models	Label_Ratio						
		0.1	0.2	0.3	0.4	0.5	0.6	0.7
Supervied regression	kNN	13.37	14.33	12.71	11.95	10.31	**9.04**	**9.55**
	SVR	15.56	16.31	16.31	16.18	15.89	15.49	15.41
	ET	15.29	15.28	12.79	14.80	18.32	13.57	20.52
	GPR	15.38	15.73	15.51	15.62	15.25	15.02	15.12
SSR	Coreg-kNN	**12.77**	**12.33**	11.85	**11.14**	**9.82**	9.86	9.82
	Coreg-SVR	15.56	16.31	16.31	16.18	15.90	15.49	15.41
	SSDKL	15.88	15.95	**10.74**	14.43	15.26	13.93	14.35
AL	SSDKAL	15.39	14.44	13.82	11.38	12.71	12.31	10.26

**Table 7:** Time Consumption for Different Regression Models based on Mode I.

Model Class	Models	Label_Ratio						
		0.1	0.2	0.3	0.4	0.5	0.6	0.7
Global features	kNN	0.00	0.00	0.00	0.00	0.00	0.00	0.00
	SVR	0.00	0.00	0.016	0.016	0.016	0.031	0.031
	ET	0.00	0.00	0.00	0.004	0.00	0.00	0.00
	GPR	0.016	0.016	0.016	0.031	0.028	0.063	0.062
Supervied regression	kNN	0.016	0.00	0.007	0.00	0.00	0.00	0.016
	SVR	0.016	0.00	0.016	0.016	0.031	0.031	0.040
	ET	0.00	0.016	0.016	0.00	0.016	0.022	0.020
	GPR	0.00	0.031	0.016	0.047	0.069	0.100	0.127
SSR	Coreg-kNN	15.32	7.70	21.70	4.44	8.52	25.64	32.21
	Coreg-SVR	8.53	5.12	8.27	35.78	18.24	53.17	42.67
	SSDKL	6.68	12.32	20.29	27.63	34.60	41.64	50.66
AL	SSDKAL	**14.30**	**29.13**	**41.37**	**54.21**	**68.83**	**82.63**	**96.73**

**Table 8:** Results of ablation experiments based on Mode I.

Models	Label_Ratio						
	0.1	0.2	0.3	0.4	0.5	0.6	0.7
GPR	41.83	41.71	42.33	42.41	42.41	41.94	41.69
DNN	53.93	45.55	58.77	42.48	44.37	43.01	43.03
DKL	40.45	27.71	22.05	17.75	14.90	13.96	9.06
SSDKL	20.53	20.03	13.44	12.51	13.21	11.67	9.75
SSDKAL	**15.90**	**14.10**	**10.10**	**8.33**	**8.84**	**9.22**	**7.86**

**Table 9:** Results of different AL query strategies and different kernels based on Mode I.

AL Query Strategy	Kernel	MSE
QBC + GS	RBF	**10.10**
	Cosine	41.01
	Matern	13.80
	RQ	13.61
QBC + GS	RBF	**10.10**
QBC		12.61
GS		11.99
QBC Group		12.24

**Table 10:** Results of different AL query strategies and different kernels based on Mode II.

AL Query Strategy	Kernel	MSE
QBC + GS	RBF	**11.85**
	Cosine	23.32
	Matern	12.47
	RQ	13.16
QBC + GS	RBF	**11.85**
QBC		13.75
GS		16.41
QBC Group		13.15

**Table 11:** Comparison of the proposed method with existing methods.

Methods	Mode I	Mode II
Preston model [45]	870.25	
Reconst. + Clust.loss [15]	32.34	
Luo & Dornfeld model [45]	288.08	
FE12 + CR [12]	312.915	307.091
CART-Stacking [6]	25.65	20.25
ELM-Stacking [6]	22.99	20.12
kNN-MTGP [7]	9.94	13.20
JIT-PF [8]	9.88	13.32
SSDKAL	**7.86**	**9.95**

## Data Availability

Not applicable.
